# Supplementary material for: Phylogenetic, Evolutionary and Structural Analysis of Canine Parvovirus (CPV-2) Antigenic Variants Circulating in Colombia
Source: Viruses. 2020 Apr 30;12(5):500. doi: 10.3390/v12050500 (PMC7290427; doi:10.3390/v12050500)
Supplement: Supplementary file 1 [file viruses-12-00500-s001.pdf]

Phylogenetic, evolutionary and structural analysis of canine parvovirus (CPV-2) antigenic variants circulating in Colombia

Sebastián Giraldo-Ramirez, Santiago Rendon-Marin and Julián Ruíz-Saenz *

Grupo de Investigación en Ciencias Animales - GRICA, Facultad de Medicina Veterinaria y Zootecnia, Universidad Cooperativa de Colombia, sede Medellín

* Correspondence: Calle 30A # 33-51, Universidad Cooperativa de Colombia, Bucaramanga, Colombia. Tel: +57-7-685 45 00 ext. 7072, *E-mail address*: julianruizsaenz@gmail.com; julian.ruizs@campusucc.edu.co (J. Ruiz-Saenz) **ORCID ID:** 0000-0002-1447-1458

Supplementary Table 1. Sites under positive selection in Colombian CPV-2a and CPV-2b variants.

**site**	**α**	**β**	**B-α**	**Prob [α> β]**	**Prob [α< β]**	**Bayes factor [α< β]**
297	2.248	27.004	24.756	0.014	0.965	48.831
324	3.262	34.508	31.245	0.017	0.957	39.451
426	3.354	22.649	19.295	0.038	0.928	22.968
440	1.551	19.874	18.323	0.013	0.968	53.932

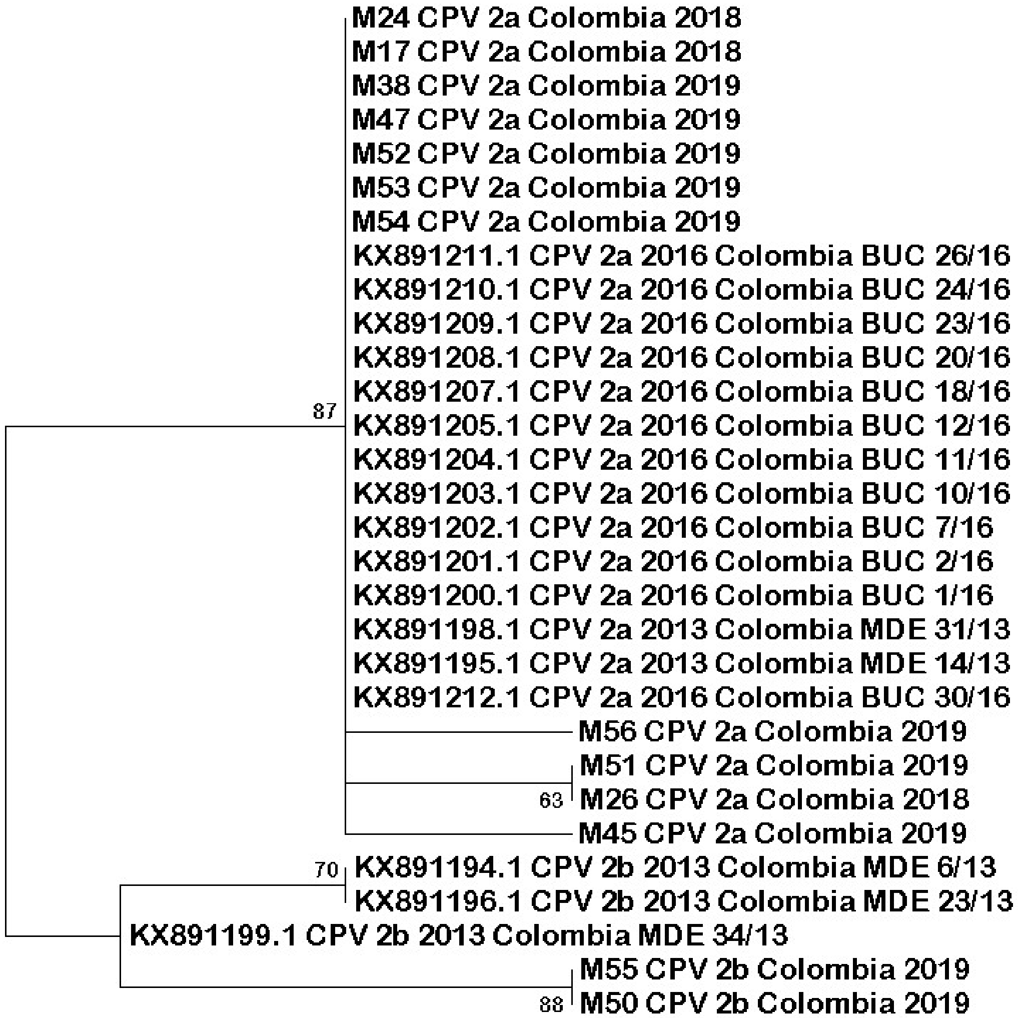

Supplementary Figure 1. Partial VP-2 Maximum likelihood phylogenetic analysis of Colombian CPV-2 sequences (Bootstrap 1000 - Tamura 3 parameter model). Current sequences denote as Colombia 2019.

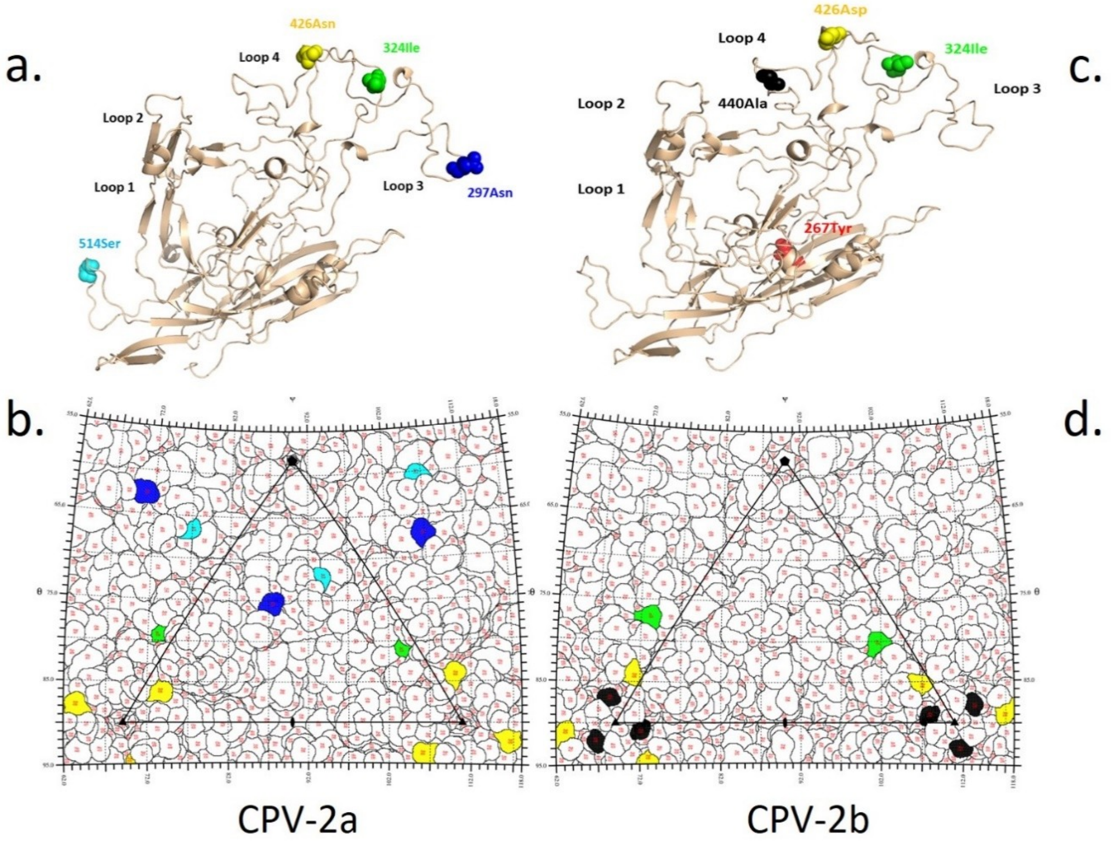

Supplementary Figure 2. Three-dimensional reconstruction and structural analysis of the VP2 surface of the Colombian CPV-2a and CPV-2b variants. (a) VP2 tertiary structure model of CPV-2a. The image shows the mutations detected in the sequence analysis; 297Asn (blue) and 324Ile (green) are located in the loop 3, an exposed area of the VP2 structure. The amino acid 514Ser (light blue) is located in an area of less exposure. (b) Surface representation of VP2. The superficial location of the amino acids indicated in the previous image with the same colours is demonstrated. The 297Asn, 324Ile and 514Ser mutations are within the VP2 region that is involved with the interaction with the TfR receptor and antibody neutralization zones (Voorhees et al., 2019). (c) Model of the VP2 tertiary structure of CPV-2b. The mutations observed in the sequence analysis are illustrated; 267Tyr (red) is located in the inner face of the VP2 protein. The amino acid 324Ile (green) is observed in the loop 3, similar to that in the CPV-2a variant, whereas 440Ala (black) is detected in the loop 4, the most prominent region of the structure. (d) Representation of the VP2 surface of CPV-2b. The topological location of the mutations indicated in the previous image is shown. Because 267Tyr is observed on the inner side of the protein, it is not represented in the image. The representation reveals the proximity between 440Ala and 426. This threefold axis is the site of greatest antigenic importance of the virus, and 426 is the main antigenic determinant of the virus. In all the images, the amino acid 426 (yellow) is represented as the reference of the structure.
